# Global-scale seasonally resolved black carbon vertical profiles over the Pacific

**DOI:** 10.1002/2013GL057775

**Published:** 2013-10-23

**Authors:** J P Schwarz, B H Samset, A E Perring, J R Spackman, R S Gao, P Stier, M Schulz, F L Moore, Eric A Ray, D W Fahey

**Affiliations:** 1 Chemical Sciences Division, Earth System Research Laboratory, National Oceanic and Atmospheric AdministrationBoulder, Colorado, USA; 2 Cooperative Institute for Research in Environmental Sciences, University of ColoradoBoulder, Colorado, USA; 3 Center for International Climate and Environmental Research – Oslo (CICERO)Oslo, Norway; 4 Science and Technology CorporationBoulder, Colorado, USA; 5 Atmospheric, Oceanic and Planetary Physics, Department of Physics, University of OxfordOxford, UK; 6 Laboratoire des Sciences du Climat et de l'EnvironnementGif-sur-Yvette, France; 7 Global Monitoring Division, Earth System Research Laboratory, National Oceanic and Atmospheric AdministrationBoulder, Colorado, USA

**Keywords:** black carbon, aerosol, AeroCom, tropical tropopause layer, HIPPO, remote

## Abstract

[1] Black carbon (BC) aerosol loadings were measured during the High-performance Instrumented Airborne Platform for Environmental Research Pole-to-Pole Observations (HIPPO) campaign above the remote Pacific from 85°N to 67°S. Over 700 vertical profiles extending from near the surface to max ∼14 km altitude were obtained with a single-particle soot photometer between early 2009 and mid-2011. The data provides a climatology of BC in the remote regions that reveals gradients of BC concentration reflecting global-scale transport and removal of pollution. BC is identified as a sensitive tracer of extratropical mixing into the lower tropical tropopause layer and trends toward surprisingly uniform loadings in the lower stratosphere of ∼1 ng/kg. The climatology is compared to predictions from the AeroCom global model intercomparison initiative. The AeroCom model suite overestimates loads in the upper troposphere/lower stratosphere (∼10×) more severely than at lower altitudes (∼3×), with bias roughly independent of season or geographic location; these results indicate that it overestimates BC lifetime.

## 1. Introduction

[2] The remote regions of the atmosphere at high altitude, and far from population centers, act as a sink for many atmospheric constituents sourced in polluted areas. For black carbon (BC) aerosol, an important climate forcing agent [*Bond et al*., 2013], the largest uncertainties in predicting its atmospheric abundance in these regions are associated with modeling its removal [*Vignati et al*., 2010]. Measurements far from sources can strongly constrain model representations of this important process [*Koch et al*., 2009; *Kipling et al*., 2013] but are presently too sparse; wider temporal and spatial coverage and improved statistics are needed. BC has unique features as a tracer that gives it additional value that has not yet been fully realized. These include the fact that it is almost solely removed from the atmosphere via precipitation because it is chemically stable and generally contained in small enough particles to avoid significant gravitational settling, it has no significant sources in the remote atmosphere explored here, and it can be detected even in very low concentration.

**Figure 1:**
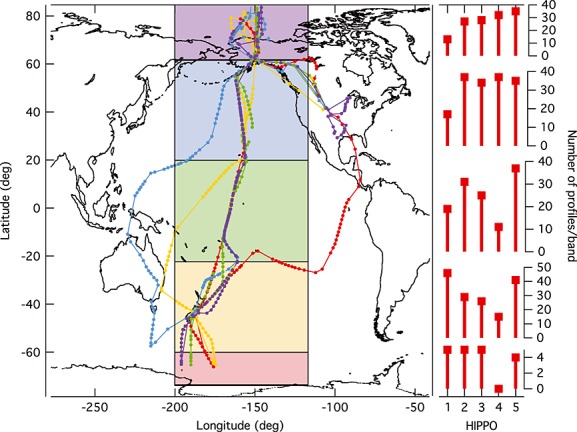
Map showing the flight track of the NSF/NCAR GV on HIPPO series 1–5 (HIPPO 1: January 2009, red; HIPPO 2: November 2009, orange; HIPPO 3, March/April 2010, green; HIPPO 4, June 2011, blue; HIPPO 5, August, 2011, purple). The markers represent the central location of each vertical profile and are only included for profiles with at least two valid 1 km altitude average BC MMR values. The colored bands show the regions averaged for comparison to models. On the right axes are shown the number of profiles in each latitude band from each HIPPO flight set.).

[3] Here we present a data set of airborne in situ measurements of BC aerosol mass mixing ratios (MMRs) obtained via extensive vertical profiling of the remote atmosphere over global scales, with comparison to models. The measurements were collected as part of the High-performance Instrumented Airborne Platform for Environmental Research (HIAPER) Pole-to-Pole Observations (HIPPO) research aircraft campaign [*Wofsy et al*., 2011, 2012] with a single-particle soot photometer (SP2, Droplet Measurement Technology, Inc., Boulder, CO) during five 3 week measurements series mainly over the Pacific (Figure 1). The aircraft flew while near continuously vertically profiling the atmosphere from a few hundred meters altitude up to ∼8 km (with less frequent climbs to ∼14 km) above mean sea level. Analysis of the first one fifth of the data set was presented previously and has proved valuable in evaluating model performance both in climatological [*Schwarz et al*., 2010] and nudged comparisons [*Fan et al*., 2012]. The full remote data set presented here dramatically extends that initial work to provide seasonal information, improved statistics, and a BC climatology that permits identification of features that are suitable for constraining models via focused studies. This data set also reveals the utility of BC MMR as a tracer of large-scale circulation in the atmosphere.

[4] The HIPPO BC climatology is compared here to model predictions generated as part of the AeroCom project (http://aerocom.met.no/). AeroCom was initiated to allow evaluation and comparison of global aerosol models using a shared data protocol and emissions inventory [*Myhre et al*., 2013], thereby permitting the underlying causes of intra-model disparities to be explored while minimizing the sources of the disparities. A “Phase I” model run conducted in 2004 [*Schulz et al*., 2006] produced results that were previously compared to the first one fifth of the data included here [*Schwarz et al*., 2010]. New “Phase II” results using updated models are used now. An AeroCom Phase I estimate of BC direct forcing formed much of the basis for the most current estimate of BC's total radiative forcing [*Bond et al*., 2013] yet was adjusted significantly based on comparison to sun photometer surface measurements of absorption AOD and the previous HIPPO intercomparison. The large uncertainty of these adjustments warrants further evaluation of large-scale AeroCom ensemble features, which we present here.

[5] Description of the data set analysis and model comparison approach is presented in section 2. In section 3, the BC vertical profiles are presented with discussion about their relevant features and application to estimating the extratropical influences on the tropical tropopause layer (TTL) region. In section 4, the model ensemble/measurement comparison results are presented, and in section 5, we summarize our findings and their significance.

## 2. Measurements and Analysis

[6] A single-particle soot photometer (SP2) was used to detect BC mass in individual particles. Adopting the nomenclature recommended by *Petzold et al*. [2013], we refer to the SP2-measured material as “refractory black carbon” (rBC). The rBC is experimentally equivalent to elemental carbon (EC) measured using thermo-optical analysis [*Kondo et al*., 2011], which forms the basis for the AeroCom BC model inventory. Additional information about the details of the setup and uncertainties of the SP2 in HIPPO can be found in *Schwarz et al*. [2010] and in the supporting information. The total systematic measurement uncertainty (30%) is negligible in the comparison here, which shows dramatic model bias with respect to the measurements. The statistical uncertainty in the rBC MMR measurements arises primarily from counting statistics limitations and varies with rBC MMR and size distribution, aircraft altitude, ambient temperature, and integration time (details included in the supporting information). Here for a single 2 min integration, statistical detection limit varied from 0.03 ng rBC/kg air at low altitude to at worst 0.3 ng rBC/kg air at the highest altitudes. Generally, we have a sufficient quantity of measurements within the various regions of analysis (below) that statistical uncertainty within each region is limited not by instrument uncertainty but by the variability in the air masses sampled.

[7] Figure 1 shows a map with the ground tracks of the five flight series that were carried out at different times of the year by the HIPPO aircraft (the NSF/National Center for Atmospheric Research (NCAR) GV, formerly known as the HIAPER). The GV performed most vertical profiles from ∼0.3 to ∼8 km altitude, yet after the take off for each flight or before arrival at the destination airport, air traffic control would often permit higher-altitude legs up to the practical aircraft ceiling of ∼ 14.5 km altitude (Figure S2 in the supporting information).

[8] The rBC MMR measured in cloud-free air was averaged into 1 km high-altitude bins (at the climb/descent rate of the aircraft, each corresponding to approximately 2 min of flight) to generate an independent vertical profile for each ascent or descent of the aircraft, as in *Schwarz et al*. [2010]. For short periods between ascents and descents, the aircraft flew level legs, typically at 8 km altitude. For these portions of flight, the data was split evenly between the initial-ascent and following-descent profiles, improving instrument statistics at this altitude level. Occasionally, level legs of longer duration (>10 min) were flown (almost always near the ceiling of the aircraft's altitude range; Figure S2). In these cases, 5 m of data from the end of the ascent or 5 m of data before the start of the descent were also included in the relevant profile, further improving the statistical significance of data at the top of the highest profiles.

[9] The individual vertical profiles generated in this manner were treated as the basic unit of comparison to avoid the possibility of bias between the model and observational results due to non-regular flight patterns and noncontinuous data. Hence, profiles were averaged together with equal weight in each altitude bin independently of the number of SP2 rBC MMR values that contributed to each bin. Figure 1 shows the number of profiles obtained in each of the five latitude bands for each flight series. Of the ∼750 total profiles, 34 profiles had no rBC data due to cloud and local pollution cuts or technical problems and hence are not included in the analysis here. Approximately 700 had at least two values; ∼600 had at least 6 valid 1 km altitude bin values.

[10] The AeroCom Phase II suite of models is described in *Myhre et al*. [2013, and references therein]. Briefly, the NCAR-CAM3.5, CAM4-Oslo, CAM5.1, GISS model E, GMI, GOCART, HADGEM2, IMPACT, INCA, ECHAM5-HAM, OsloCTM2, and SPRINTARS models produced BC MMR fields with resolutions varying from 1.1° × 1.1° to 5.0° × 4.0° (average = 2.6° × 2.0°) and between 19 and 72 vertical levels (average = 40). Emission inventories used were for the year 2000. From each of the 12 models of this suite, vertical profiles of BC MMR were extracted at the grid point locations closest to the central latitude/longitude of each vertical profile of the aircraft, for the appropriate month of the year (model simulations were climatological or nudged to the year 2006). Uncertainties in the comparison arising from these manipulations are minor (supporting information). After extraction, the model profiles were interpolated to the same vertical grid upon which the measured profile data set was averaged and only generated for altitude bins for which valid SP2 data contributed to the corresponding measured profile. Individual model profiles were then grouped as an ensemble and averaged in different latitude bands to provide mean and 25th/75th percentile values from the model ensemble. The measured profiles were treated in the same way to provide corresponding averages.

## 3. Black Carbon Vertical Profiles

**Figure 2:**
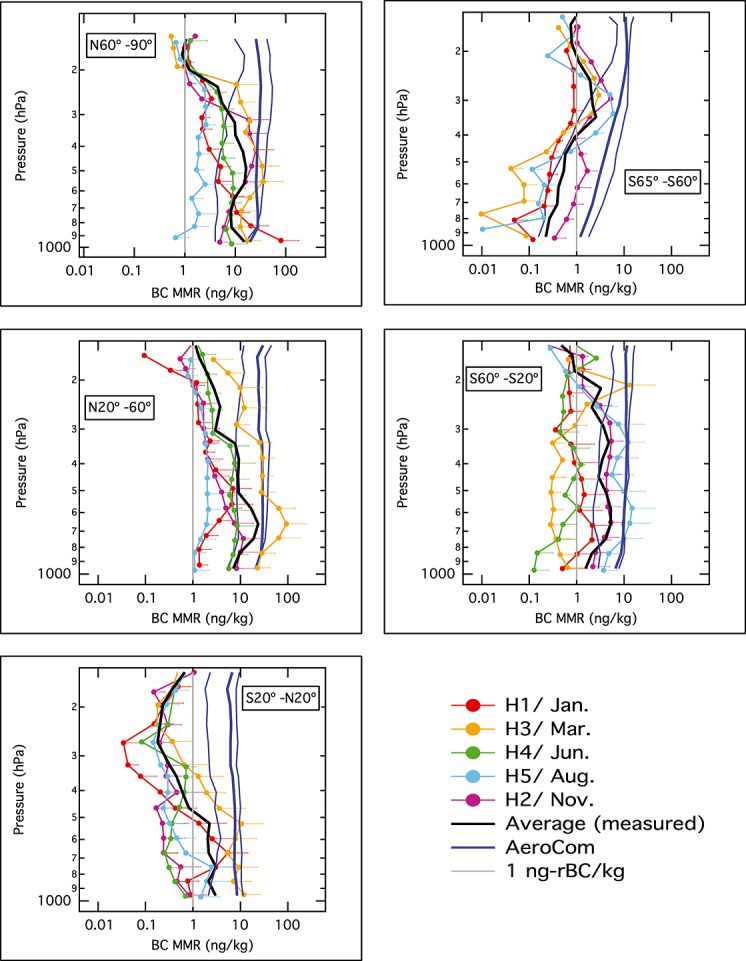
Measured and modeled vertical BC profiles in five latitude bands. The measured profiles are colored from red to purple throughout a calendar year, with the thick black line providing their average (and an approximate annual average). The AeroCom model suite average profile is the thick darker blue line, with 25th and 75th percentile values given by the thin darker blue lines. Shown in grey, as a guide to the eye, is 1 ng rBC/kg mass mixing ratio.

[11] Figure 2 summarizes the rBC measurements, revealing climatological rBC MRR in the remote atmosphere averaged over five broad latitude bands: 67–60°S, 60–20°S, 20°S–20°N, 20–60°N, and 60–85°N, in a longitude band (200–120°W) far from significant pollution sources. Each panel presents a different latitude band, with the colored lines showing average profiles from the individual HIPPO series (hence the various seasons), and the heavy black line showing the average over all five HIPPO series (hence an approximate annual average). Whiskers represent statistical variability, which is predominantly representative of intra-profile variability within the latitude band of each series. The statistical uncertainty in the approximate annual averages can be estimated by eye from the intra-seasonal variations. Several prominent features immediately stand out: the reduction of seasonal variability with convergence to MMR near 1 ng rBC/kg in the high altitudes of all the latitude bands is most obvious in the northern 60°N–85°N band; the higher rBC MMR throughout the column in spring compared to other seasons in the 20°N–60°N region; and the quite consistent lack of strong seasonal variability in rBC MMR around values of ∼ 0.2 ng rBC/kg at ∼200 hPa in the upper tropical troposphere region (20°S–20°N), increasing with altitude to match the values seen at the highest altitudes aloft except in the mid-northern band (∼1 ng rBC/kg). These climatological features are discussed in more detail below.

[12] The stability of the rBC MMR in the northern lower stratospheric region is clearly notable and very compelling for comparison to models. Over the 3 calendar years of the HIPPO campaign, the average rBC MMR in this region fell in the range 0.5–2 ng rBC/kg for all seasons for air masses containing up to 1500 ppb of ozone. Examination of N_2_O-SF_6_ tracer-tracer relationships for the high-altitude polar (both northern and southern) air masses indicates that this air was predominantly stratospheric from a tropical injection point [*Ray et al*., 1999] (Figure S3). Hence, this stability is likely due to long time scales of mixing and transport into and from the tropical tropopause region (at least ∼1 year) and likely constrains rBC MMR variability in the tropics at 380 K potential temperature, the isentrope defining the bottom of the stratosphere. It follows that rBC loadings throughout the lower stratosphere (which are driven by tropical input, as there are no known significant stratospheric sources of BC) are likely constant in this range around 1 ng rBC/kg. This is supported by the observation that rBC MMR in the tropics appears to increase with altitude toward this value without significant variability in the seasonal averages; a feature that is discussed next.

**Figure 3:**
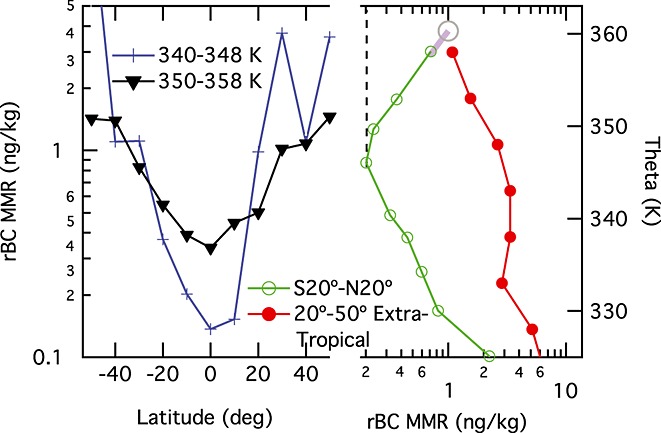
Trends in rBC MMR across the TTL. (left) The rBC MMR from the entire HIPPO data set at two potential temperature (theta) ranges is plotted against latitude. (right) The vertical profile of rBC MMR for the tropics (as in Figure 2) is plotted against potential temperature with the extratropical profile generated by averaging the profiles from south and north 20°–50°. The tropical profile and its minimum are extrapolated to 1 ng rBC/kg at 360 K, as represented by the gray extension and circled point, and the dashed line, respectively.

[13] In the equatorial region (20S–20°N), the cleanest air is seen in the 200–300 hPa pressure range, with rBC MMR increasing above quite consistently in each season and with clear statistical significance in the approximate annual average. This feature is likely due to injection of BC-scrubbed air via convection being balanced with latitudinal mixing from the extra tropics into the tropical tropopause layer (TTL). Convective outflow in the tropics peaks at roughly 200 hPa [*Folkins and Martin*, 2005], largely consistent with the observed annual average minimum in rBC MMR, while the increase above the minimum is expected due to horizontal (isentropic) exchange of air with the extra tropics out to 50° latitude [*Konopka et al*., 2007]. Figure 3 shows that rBC MMR has large gradients between the extra tropical and tropical upper troposphere; these gradients are much larger than those observed in the trace gas ozone that has been used previously to infer isentropic mixing of extratropical air into the TTL along contours of constant potential temperature (theta) [e.g., *Konopka et al*., 2007]. This new observation suggests that the strong sensitivity of TTL BC MMR to horizontal mixing can be used to constrain the amount of extra tropical air that was mixed into the tropics. Based on simple analysis (expanded in the supporting information) of the average tropical (0–20° latitude, both hemispheres) and extra tropical (20–50° latitude, both hemispheres) rBC profiles shown in Figure 3, we estimate that the percentage of air mixed into the TTL from the extra tropics ranges from roughly 5% at the 350 K theta contour up to 55% near the 360 K contour. These mixing fractions are believed to be lower bounds because the observed minimum rBC MMR value in the tropics (at ∼200 hPa; Figure 2) is likely higher than it would be in the absence of mixing. Our mixing estimate near 360 K theta is consistent with calculations done using a global model [*Konopka et al*., 2007]. Note that the latitudinal gradients of black carbon shown in Figure 3 suggest that the mixing from the extra tropics does not uniformly extend to the equator and that at least the lowest part of the TTL is not fully mixed.

[14] In the northern midlatitudes (20–60°N), the influences of Asian outflow can be seen in the rBC MMR elevated with respect to other seasons throughout the column in spring. This was measured near the regular peak season of annual Asian influence on the Pacific. Here, for pressure altitude above ∼250 hPa, we observe the largest variability in measured rBC MMRs of all the latitude bands explored. This likely indicates the strong Northern Hemisphere sources that contribute over wide longitude ranges to the loadings but is also due, in part, to relatively poor profile statistics in HIPPO 1 (Figure 1), which provides the lowest average rBC MMR here; the single highest altitude HIPPO1 rBC MMR value in this latitude band is based on two profiles, while the next highest was based on four.

## 4. AeroCom Phase II Comparison

[15] Figure 2 includes the AeroCom ensemble mean and 25th/75th percentile vertical profiles averaged over all five HIPPO flight series. Independent of latitude band, the ensemble shows only weak dependences of BC MMR on the vertical dimension in the HIPPO regions analyzed here. Only in the southern polar region does the ensemble have a clear vertical dependence, tracking the positive correlation between altitude and BC MMR that is also present in the measurements up to pressures of ∼300 hPa. In seasonally resolved measurements (i.e., on individual HIPPO series comparisons within each latitude band; not shown), the AeroCom ensemble performance has much the same character. Note that as 12 models are used, the 75th and 25th percentile lines trace the third lowest and third highest individual model results at each pressure level. The fact that these also show little vertical dependence reveals that many of the individual AeroCom models output BC MMR fields that share this property in the annual average profiles.

**Figure 4:**
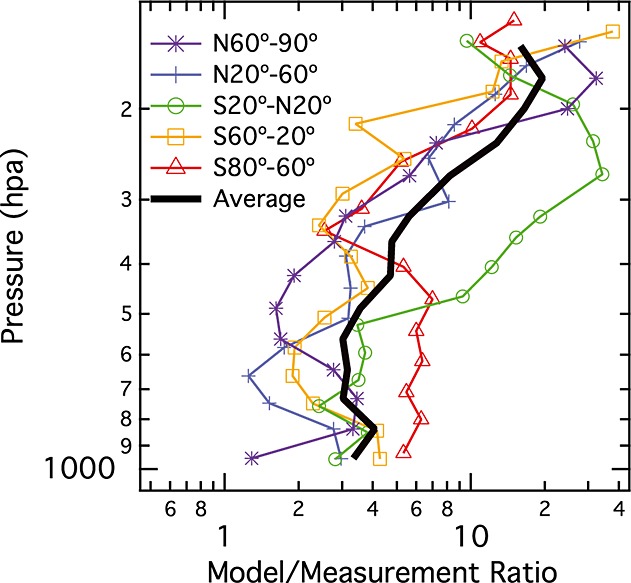
The ratio of the modeled to measured vertical BC profiles for individual latitude bands (colored lines) and averaged overall latitudes (black line). These results are averaged over all seasons/HIPPO series.

[16] As the measurements tend to show significant reductions in rBC MMR aloft, the model ensemble performance degrades at high altitude. Figure 4 shows the ratio of the AeroCom model results to the measured rBC MMR vertical profiles for the full HIPPO data set in each latitude band (i.e., the model annual averages (thick blue lines) divided by the measured annual averages (thick black lines) from Figure 2). The model/measurement ratio averaged over all five latitude bands is also shown. On average, the ensemble is a factor 3 high below 500 hPa, a factor 7 high for 500–250 hPa, and a factor 17 high for 230–150 hPa. In column loading, the model average bias ranges from a factor ∼ 2.5–6, with an average factor of 4 over prediction.

[17] The model ensemble vertical profile's lack of BC MMR dropoff in the convective outflow region of the tropics is responsible for the particularly large bias in the pressure range 500–200 hPa in this region, where the measurement shows reduced loadings. In general, focused studies are necessary to identify the model factors that contribute to various features of vertical structure, yet here the data clearly show that the dominant impact of tropical convection is in net removal of BC. This is the obvious explanation for the approximately tenfold reduction in BC MMR from the convective inflow altitudes to its outflow altitudes. Hence, in the tropics, it appears that convective removal is underrepresented by the model ensemble. Recent work using the HadGEM2-UKCA model showed that an improved representation of convective scavenging greatly improved that model's performance in the tropical upper troposphere [*Kipling et al*., 2013].

[18] The AeroCom ensemble bias at high altitude in the near-polar latitude bands, and close to the 380 K isentrope in the tropics, suggests that the errors due to underestimated removal in the tropics could be carried through the lower stratospheric circulations. Note that the model setups used in the AeroCom submission generally do not provide enough resolution and vertical extent to resolve stratospheric circulations. The fact that the bias persists in all the latitude bands at high altitudes with substantial zonal mixing suggests that it may extend broadly over the globe at the top of the troposphere.

## 5. Conclusion

[19] The global-scale climatology of remote BC loadings presented here provides a basis for studies of atmospheric circulation, constraint of global models, and representation of removal and transport processes. It reveals that average rBC MMRs tend to converge into the range of 1–10 ng rBC/kg in the upper troposphere, with most seasonal variability constrained below 300 hPa. Consistent rBC MMRs measured in the Northern and Southern lower stratosphere show a very narrow range of 0.5–2 ng rBC/kg, matching the loadings from the highest-altitude measurements in the tropics. This surprising feature serves as an excellent boundary condition for testing, evaluating, and tuning global models of BC.

[20] The rBC MMR has gradients between the extratropical and tropical upper troposphere that are much larger than those observed in the trace gases that have been used previously to infer isentropic mixing of air into the TTL. Simple analysis of the vertical profiles of rBC in the tropics and extra tropics reveal mixing consistent with previously published values, indicating that BC has utility as a tracer in this region, and possibly in the stratosphere, that has not yet been fully assessed.

[21] Comparison of the climatology to the AeroCom Phase II suite of models indicates a high model bias throughout the column explored here. Below 500 hPa, the suite is on average a factor 3 too high, and above 500 hPa is on average a factor 11 high over all latitudes studied. The contrast of these results to evaluations of AeroCom performance near the source regions [e.g., *Bond et al*., 2013], which show low model bias, indicates that the models overestimate BC lifetime and remote transport. As BC forcing per unit mass increases by a factor ∼10 over the same altitude range [*Samset and Myhre*, 2011], the model bias for BC radiative forcing in the remote atmosphere may be even higher. The implications for reduced BC lifetime/loadings are relevant not only to the remote regions but also to the air intermediate to source regions and possibly at high altitude around the globe.
