# Supplementary material for: Global-scale seasonally resolved black carbon vertical profiles over the Pacific
Source: Geophys Res Lett. 2013 Oct 23;40(20):5542–7. doi: 10.1002/2013GL057775 (PMC4542199; doi:10.1002/2013GL057775)
Supplement: Supplementary file 1 [file grl0040-5542-sd1.docx]

Auxiliary material for

Global-scale seasonally resolved black carbon vertical profiles over the Pacific

[Paper# 2013GL057775]

J. P. Schwarz^1,2^, B. H. Samset^3^, A. E. Perring^1,2^, J. R. Spackman^1,4^, R. S. Gao^1^, P. Stier^5^, M. Schulz^6^, F. L. Moore^7^, Eric A. Ray^1,2^, and D. W. Fahey^1,2^

^1^ Chemical Sciences Division, Earth System Research Laboratory, National Oceanic and Atmospheric Administration, Boulder, CO 80305 USA

^2^ Cooperative Institute for Research in Environmental Sciences, University of Colorado, Boulder, Colorado 80309 USA

^3^ Center for International Climate and Environmental Research – Oslo (CICERO), Oslo, NO

^4^ Science Technology Corporation, Boulder, CO, 80305

^5^ Atmospheric, Oceanic and Planetary Physics, Department of Physics, University of Oxford, OX1 3PU, UK

^6^ Laboratoire des Sciences du Climat et de l'Environnement, Gif-sur-Yvette, 91191, FR

^7^ Global Monitoring Division, Earth System Research Laboratory, National Oceanic and Atmospheric Administration, Boulder, CO 80305 USA

Geophysical Research Letters

Introduction:

Supplemental information is included in the .pdf file (text01.pdf). The document includes technical information about the operation of the SP2 as well as figures and secondary discussion supporting the main manuscript.

1. text01.pdf: Contains the supplemental information
