# Supplementary material for: Global-scale seasonally resolved black carbon vertical profiles over the Pacific
Source: Geophys Res Lett. 2013 Oct 23;40(20):5542–7. doi: 10.1002/2013GL057775 (PMC4542199; doi:10.1002/2013GL057775)
Supplement: Supplementary file 2 [file grl0040-5542-sd2.pdf]

Supplemental information:

SP2 Operation and Calibration information:

Full calibrations of the rBC mass – visible thermal emission relationship for the SP2 consisted of sampling of size-selected fullerene soot (Alfa Aesar Inc., Ward Hill, MA, Lot F12S011), a well-characterized material that is the recommended calibration standard for SP2s [*Baumgardner et al.*, 2012]. The fullerene soot was size selected over a range corresponding (using the mass-to-mobility relationship of *Moteki and Kondo* [2010]) to 100 – 270 nm volume equivalent diameter (assuming void free density of 1.8 g/cc), and the calibration was very close to perfectly linear for this range.

In HIPPO 1,2,3, and 5, only one laser/detection head (the critical detection component of the SP2) was flown for the entire three-week series; the head was not adjusted over the measurement period, and was calibrated at least at the beginning and end of each flight series. Under these conditions, we saw excellent (~5%) stability in calibrations of SP2 rBC mass sensitivity. In HIPPO4, the optical head lost laser power during the Southern-most legs of that flight series. A spare head (including laser and optical detectors) was installed and flown for the rest of the mission. Both the initial flight head and the spare head were fully calibrated before the HIPPO4 flight series began. Based on the calibration stability observed on the other flight series, we only use the calibration on these two heads for the start (on the initial flight head) and end (on the spare head) of the series.

In addition to the fullerene soot, each calibration included measurements of 220 nm polystyrene latex spheres, to directly measure the laser intensity, as described in *Schwarz et al*. [2010b]. Polystyerene latex spheres were also sampled throughout the flight series, typically before or after each flight. Laser intensity was always maintained at a high enough level to ensure proper detection of accumulation mode BC mass (as per *Schwarz et al.* [2010b]).

SP2 detection was limited to a particle size range that typically covered ~90% of the single observed accumulation mode of rBC mass, hence the observed rBC MMR was scaled upwards by 10% to represent total accumulation mode BC as in Schwarz et al. [2010]. We associate a total systematic uncertainty on rBC MMR determined with the SP2 of ±30% due to this type of manipulation coupled with additional calibration uncertainties (discussed in the supplemental material). Note that the SP2 is very insensitive to non-rBC mass, and hence does not conflate the mass of other absorbing species, such as mineral dust, with that of rBC.

As the SP2 integrates the rBC mass of individually detected particles to generate an rBC mass-mixing ratio (MMR, ng/kg) or an rBC concentration (ng m^{-3}), its statistical uncertainty in relatively clean air is dominated by counting-statistics and hence a strong function of integration time. We have used a Monte-Carlo approach to quantify this counting statistics limit, which contributes random scatter to the measurement, but does not introduce systematic bias in the results.

Random sets of rBC masses with the geometric median mass diameter and log-normal widths characteristic of rBC in the remote atmosphere (from Schwarz et al., 2010) were generated in different volumes of sample air using the "lognormalnoise" function of IGOR (Wavemetrics, inc.). An "SP2-observed" concentration was then generated by integrating the mass of only the particles within the detection range of the SP2 in HIPPO (90-550 nm rBC volume-equivalent diameter assuming 1.8 g/cc density). At different modeled concentrations, a set of different volumes of sample air was tested each on 100 different runs, and the standard deviation in the SP2-observed value was determined for each volume. For the particular setup of the SP2 in HIPPO, Figure S1 shows the relationship between integration time and the statistical uncertainty.

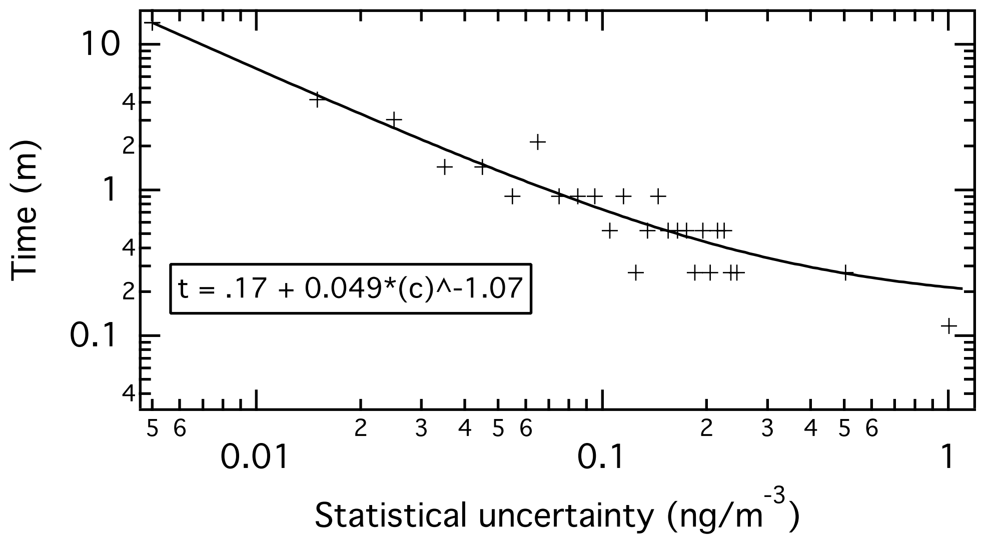

Figure S1: SP2 statistical uncertainty related to integration time as determined by monte-carlo analysis for a population with the BC mass size distribution typical for the remote

atmosphere (from Schwarz et al., 2010), SP2 detection range as in HIPPO, and 4 cm^3 s^{-1} sample flow rate

For the approximate integration time for a single 2 minute integration used in the analysis below, at the operating conditions of 4 volumetric cm^3 s^{-1} sample flow and for the rBC particle size distribution observed during HIPPO [Schwarz et al., 2010], the statistical variability is 0.03 ng-rBC/m^3. The statistical uncertainty associated with rBC mass-mixing ratio (rather than volumetric concentration) requires the volume-flow rate of air sampled into the instrument to be converted to a mass-flow rate. This conversion depends on ambient temperature and altitude, and aircraft/instrument temperature, and causes the statistical uncertainty in MMR to vary from 0.03 near the ground to 0.3 ng/kg at the highest altitudes (still for a single 2 m average).

In rare occasions when aircraft GPS failed, altitude was determined from ambient pressure interpolated to the altitude/ambient pressure relationship from nearby profiles.

Data selection:

Data collected in both liquid and ice clouds were removed using a combined data set from cloud probe instrumentation on the aircraft and assessment of particles detected by the SP2 and associated with abraded inlet material (e.g. *Perring et al.*, 2013). Additionally, fresh pollution observed within a vertical range of 2 km on

approaches/departures from airports was removed to preserve the remote nature of the dataset and avoid biases associated with the logistical requirements of the mission.

Availability of high-altitude profiles:

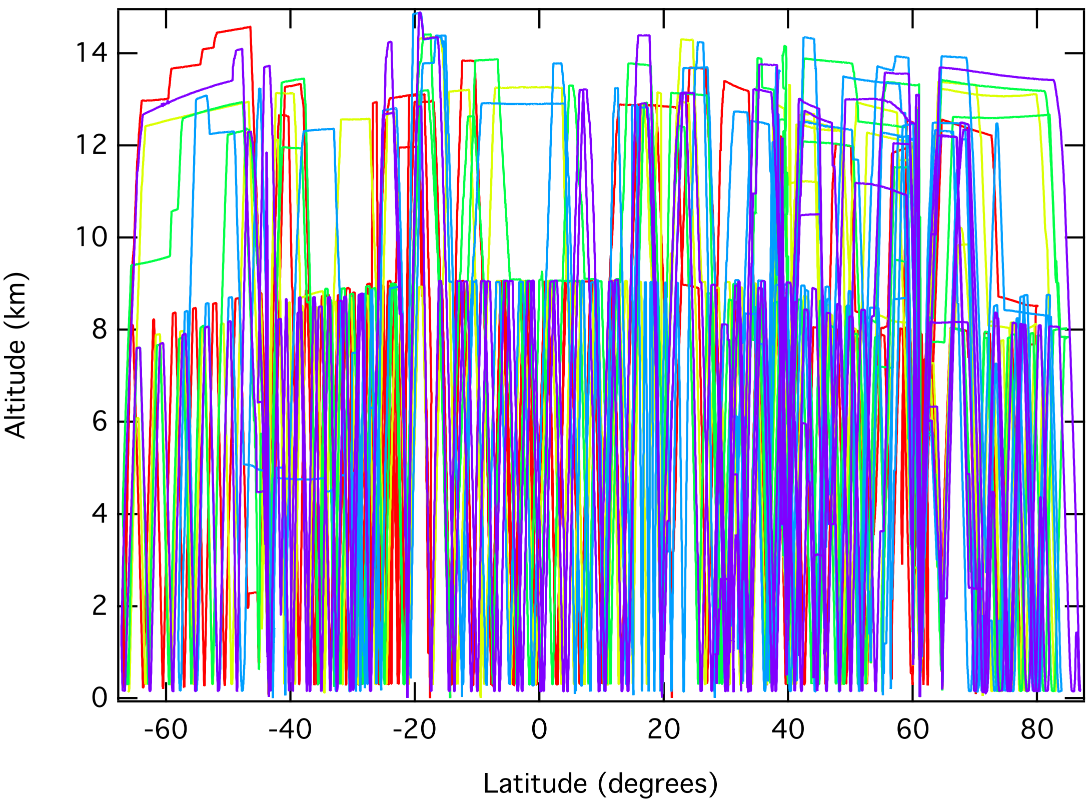

Figure S2: The altitude/latitude path of the NSF/NCAR GV aircraft over all five HIPPO series. This figure is meant to convey the relative frequency of high altitude (~14 km) profiles to the standard ~8 km profiles. Color scheme matches that in Figure 1 of the main text. The level-leg altitudes vary with latitude because the aircraft autopilot controls to constant pressure altitude, here GPS altitude is shown.

Approximations in the model/measurement comparison:

Model values were extracted at the model grid points closest to the measured profile positions. Hence, depending on model grid resolution, there could be an error of up to 2.5 degrees between the sample location and the modeled location, as well as at most a 2-week difference between the (quasi-climatological) measured and modeled periods. The models showed only weak latitude dependence from one grid point to the next, and relatively weak temporal dependence at the time of year of the largest possible time difference, so these approximations are reasonable. As the average values of rBC vertical profiles cover wide ranges of latitude typically over 1 – 2 weeks, these differences are negligible in the context of the measurement/model comparison (note that the median plume scale for HIPPO 1-3 along the flight track was only 113 km [*Weigum et al.,* 2012]), and especially for the approximate annual average results based on averaging all five HIPPO series.

Polar stratospheric tracer-tracer relationship:

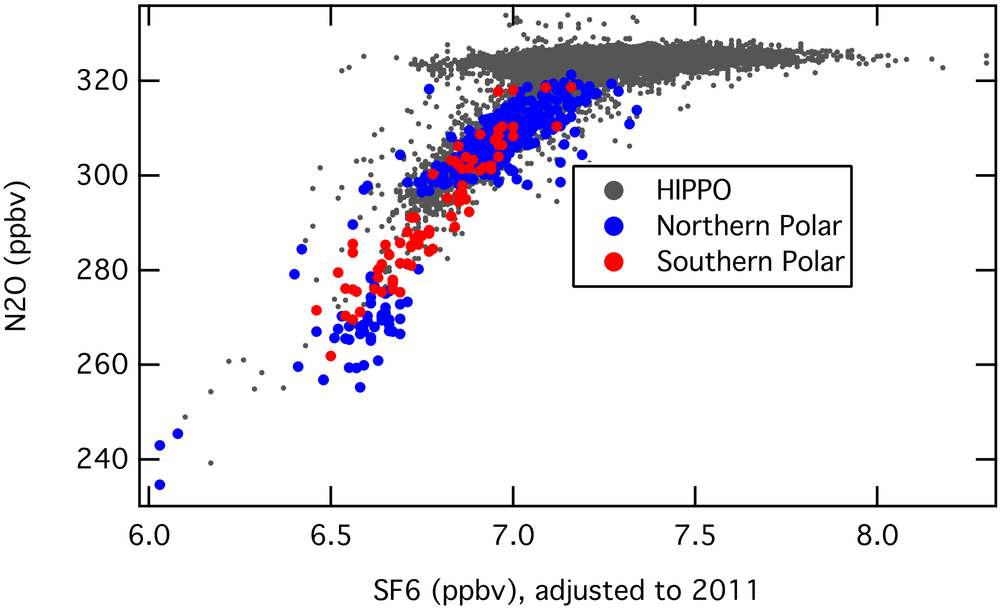

Figure S3: N20-SF6 tracer-tracer relationship for the northern and southern polar high altitude measurements overlaid on the relationship for all hippo. SF6 has been corrected to 2011 levels to remove its continuing increase over the HIPPO campaign. The colored points are from N60-90 and S67-S60 above 12.5 km altitude.

Extratropical/tropical Mixing calculation:

We assume that the average measured tropical black carbon mixing ratio (χ_T) at a particular potential temperature (Fig. 3) is a mixture of convectively transported air from below (χ_C) and air mixed isentropically from the extratropics (χ_M), as shown schematically in Figure S4.

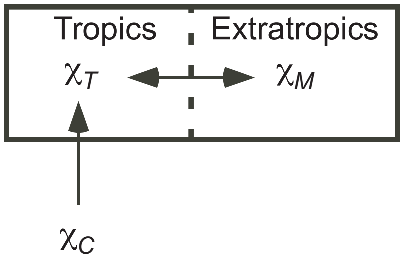

Figure S4: Simple box model for extratropical mixing above the convective outflow region.

This can be expressed as

$$\chi_T = F_M\chi_M + F_C\chi_C$$

where F_C is the fraction of air that was convectively detrained, F_M is the fraction of air mixed into the tropics from the extratropics and $F_C = 1 - F_M$. The above expression for the average tropical black carbon mixing ratio can be rearranged to solve for the fraction of air mixed from the extratropics into the tropics:

$$F_M = (\chi_T - \chi_C)/(\chi_M - \chi_C)$$

We assume that all convectively detrained air above 345 K has the minimum value of the tropical profile, so χ_C=0.2 ng/kg. We then use the profiles shown in Fig. 3 for χ_T and χ_M and solve for F_M as a function of potential temperature. For example, at $\theta \approx 355$ K, χ_M= 1.7 ng/kg and χ_T = 0.4 ng/kg and therefore F_M = 0.13. That is, the tropical black carbon mixing ratio at 355 K can be explained by a mixture of 87% air detrained from tropical

convection at the assumed minimum mixing ratio and 13% of the tropical air having been mixed isentropically from the extratropics.

There are some important caveats to the above calculation. One is that we do not have a measure of the the rBC mixing ratio of the convectively detrained air above the 350 K level. It is likely that the value is lower than the 0.2 ng/kg that we assume since deeper convection should remove more black carbon. This would mean that the above calculation is an underestimate of the fraction of air mixed into the tropics. A second caveat is that the calculation loses sensitivity at and above the level where the tropical and extratropical profiles approach 1 ng/kg. At these levels there is no longer a latitudinal gradient, so we no longer can determine from which region the tropical air originated.

Supplemental References:

Baumgardner, D. et al. (2012), Soot Reference Materials for instrument calibration and intercomparisons: a workshop summary with recommendations, *Atmos. Meas. Tech.*, **5**, 1869-1887, doi:10.5194/amt-5-1869-2012.

Moteki, N. and Y. Kondo (2010), Dependence of laser-induced incandescence on physical properties of black carbon aerosols: Measurements and theoretical interpretation, *Aero. Sci. Technol.*, **44**, 663-675.

Perring, A. E., J.P. Schwarz, R.S. Gao, A.J. Heymsfeld, C.G. Schmitt, M. Schnaiter, and D.W. Fahey, Evaluation of a perpendicular inlet for airborne sampling of interstitial submicron aerosol, *Aeros. Sci. Technol.,* **47** (10), 1066-1072, doi: 10.1080/ 02786826.2013.821196, 2013.

J. P. Schwarz, J. R. Spackman, R. S. Gao, A. E. Perring, E. Cross, T. B. Onasch, A. Ahern, W. Wrobel, P. Davidovits, J. Olfert, M. K. Dubey, C. Mazzoleni, and D. W. Fahey (2010b), The detection efficiency of the single particle soot photometer, *Aeros. Sci. Technol.,* 44:612-628, doi: 10.1080/02786826.2010.481298.
